# The effect of a gerontology nurse specialist for high needs older people in the community on healthcare utilisation: a controlled before-after study

**DOI:** 10.1186/s12877-018-0717-3

**Published:** 2018-01-22

**Authors:** A. I. I. King, M. L. Boyd, D. L. Raphael, A. Jull

**Affiliations:** 1 School of Nursing, The University of Auckland, Auckland, New Zealand; 2 School of Nursing, Faculty of Medical and Health sciences, The University of Auckland, Private Bag 92019, Auckland, Auckland Mail Centre 1142 New Zealand

**Keywords:** Older people, Gerontology nurse specialist, Primary care, Comprehensive geriatric assessment, Care coordination, Case finding

## Abstract

**Background:**

Nurse-led models of comprehensive geriatric assessment and care coordination can improve health management as well as reduce hospitalisations for high risk community dwelling older people. This study investigated the effect on healthcare utilisation of systematic case finding to identify high risk older people in the community with a subsequent comprehensive assessment and care coordination intervention by a Gerontology Nurse Specialist based in primary care.

**Methods:**

This was a controlled before-after study design located within primary healthcare practices in Auckland, New Zealand. An intervention model was initiated within two primary healthcare practices and involved a screening tool to identify high risk older people with succeeding gerontology nurse specialist assessment and care coordination. The comparison group included older people who received usual care at three comparable primary healthcare practices. The primary outcome measure was acute hospital admissions. Secondary outcomes included hospital re-admissions, length of stay, emergency department presentations, residential care admissions, and community contacts.

**Results:**

A total of 579 older people were posted the screening tool in the intervention group, with 517 completed screens (89% response rate) formulating the intervention group. A total of 101 older people were identified as high risk from these screens (20%). The comparison group comprised 883 older people. Comparing the intervention and comparison group, no statistical differences were found for hospital admissions, emergency department presentations, hospital re-admissions, length of stay, or residential care admission. Community physiotherapy showed a statistically significant increase for the intervention compared to the comparison group (*p* = 0.03). Non-significant findings revealed decreased risk of entering residential care and fewer frequent hospital re-admissions for the intervention group when compared with the comparison group.

**Conclusions:**

This specialist nurse-led intervention involving comprehensive assessment and care coordination care did not appear superior to usual care, however, there is benefit to exploring a more robust randomised controlled trial design.

**Trial registration:**

Retrospectively registered on 18/09/2017 with the Australian New Zealand Clinical Trials Registry (ANZCTR). Registration number ACTRN12617001332314

**Electronic supplementary material:**

The online version of this article (10.1186/s12877-018-0717-3) contains supplementary material, which is available to authorized users.

## Background

The population is ageing and in turn presenting a challenge for health and social care systems worldwide [1]. Older people often have complex health problems and multi morbidity which is associated high utilisation rates of health services, hospitalisation and poor quality of life [2]. For many countries there is a push for the concept of ‘ageing in place’ where older people stay in their own homes and receive home healthcare services [3]. Keeping older people in their own homes requires support from a variety of health and social services to meet their particular needs [4]. It also places an increased demand on primary care services as they attempt to cope with the demands of older adults who have complex needs [5]. To best support this distinctive sub-group, innovative models of care are required that provide the necessary crucial support. However, not all older people necessitate this type of intensive support to age in place.

An effective way of identifying high risk older people is through case finding [6]. Case finding involves screening an individual to ascertain whether they have predetermined risk factors that might lead to adverse health outcomes [7]. Once high risk older people are identified through case finding, it is essential individualised interventions are put in place to provide support and prevent worsening health or quality of life [8]. Such individualised and person centred care is recognised as crucial to holistic health care delivery [9].

Interventions which aim to manage older people in the community and prevent hospitalisation and re-admissions include care coordination and comprehensive geriatric assessment (CGA). Care coordination overcomes barriers to provide safe, integrated, quality care by connecting individuals with healthcare systems to improve health outcomes [10]. The nurse is viewed as integral to care coordination as they are well placed to ensure efficiency and effectiveness [10]. Nurse care coordination is increasingly being recognised as a method to support individuals to successfully manage their complex medical, social and psychological issues [11]. Care coordination has shown to be an effective approach to reduce hospitalisations, re-admissions and emergency department (ED) visits [12–14] as well as improve health and functional outcomes [3].

CGA involves determining an older person’s medical, psychosocial, functional, and environmental status and then devising an individualised plan for treatment and follow-up [15]. CGA has proven to be effective in improving clinical outcomes for older people as well as being cost-effective [16]. Advanced practice nurses (APN) often undertake CGA and care coordination and this position includes clinical nurse specialist roles such as the Gerontology Nurse Specialist (GNS) [17]. The CNS role signifies important leadership, education, clinical expertise and coordination components [18]. Hence, clinical Nurse Specialists (CNS) are in an ideal position to undertake CGA and care coordination and current research promotes nurse-led primary healthcare interventions to meet the needs of older people [19].

Previous models of care have not embedded a specialist nurse within the primary healthcare practice as well as strongly integrating with secondary care specialists and services. Integrated primary and secondary care involves providing improved coordination and integration of healthcare services, which is essential for health care reforms aiming to improve the quality and efficiency of healthcare services [20]. A recent systematic review found that well designed models integrating primary care and secondary care can result in positive clinical and service utilisation outcomes [21].

Using a CGA to assess individual need in conjunction with a care coordination approach to organise and deliver care may potentially improve health outcomes and reduce healthcare costs through preventing ED presentations, hospitalisations and re-admissions. A key differentiating factor of this study is that the GNS was centred within the primary care setting in addition to strong integration with secondary care. This study used systematic case finding to identify high risk community dwelling older people, who subsequently received a CGA and care coordination intervention by a GNS. The aim of this study was to evaluate the effect of this intervention on healthcare utilisation. The main hypothesis was that older people identified as high risk for health decline who received an intensive GNS intervention would have reduced hospitalisations when compared with the usual care comparison group.

## Methods

### Design

The study was a controlled before-after study design. The study period included one year before and after the intervention period; commencing October 2009 and concluding August 2013. As part of a wider study an innovative intervention model was instigated within the primary healthcare setting comprising a screening tool to identify high risk older people with succeeding GNS assessment and care coordination. The crucial innovative features of this study are that the GNS was based within the primary care setting as well as integration with secondary care. Details of implementing this innovative model with descriptive quantitative findings have been published [22] as well as qualitative perspectives of health professionals and older people [23]. This study was a pragmatic evaluation of the new model of care in relation to hospital utilisation outcomes for those who received the intervention compared with a comparable group of older people who received usual care. This study adheres to the CONSORT statement for explicit transparent reporting of clinical trials. In addition, the TIDieR guidelines and checklist [24] have been utilised to further ensure sufficient detail when describing the intervention and comparison groups see (Additional files 1 and 2).

Process evaluation for this study occurred via a project work group which was established to oversee the process of the study and provide expert guidance. This group met monthly and comprised the GNS involved in the study, key health professionals including nurse practitioners and geriatricians as well as the leading researchers involved in the study.

### Participants & setting

The intervention model of care was initiated in two primary healthcare practices. In New Zealand, primary healthcare refers to healthcare provided in the community by health professionals (including general practitioners (GP), nurses, and pharmacists) working within a general practice with the aim to maintain, enhance and restore the health of the enrolled population [25]. Independently owned primary healthcare practices are funded by a government capitation payment system (and a variety of other subsidies) as well as a partial payment per visit by patients. Not-for-profit primary health organisations (PHOs) are a consortium of primary healthcare practices established by geographical region in 2001 to ensure provision of primary healthcare services. PHOs provide primary health services either directly or through general practice members, and most primary healthcare practices are a part of a PHO. Enrolment in a PHO is voluntary, however, most New Zealanders are enrolled through their primary healthcare practice and gain the benefits associated with belonging to a PHO, which can include reduced costs for doctors’ visits and prescription medicines [25].

This study was a pragmatic evaluation where the two intervention healthcare practices identified that they wanted to implement this new model of care. The comparison group encompassed three primary healthcare practices with older people of comparable socioeconomic status, ethnicity and geographical location. All healthcare practices were located within the same PHO in Auckland, New Zealand. Older adults aged 75 years or greater and enrolled in one of the primary healthcare practices were eligible for participation in the study. Older adults were excluded if they were residing in a residential care facility at the start of the study or if at the time they were receiving care under the local hospital-based GNS team. Sample size for the intervention group was pre-determined as the two intervention healthcare practices identified they wanted to implement the new model of care. Therefore, a power calculation to determine sample size was not appropriate. To ensure meaningful comparison between the two groups, a total of three comparison group practices were selected to guarantee as a minimum the same number of participants as in the intervention group.

### Intervention

A specialist gerontology nurse-led intervention involved case finding, comprehensive assessment and care coordination. The Brief Risk Identification for Geriatric Health Tool (BRIGHT) was used to case find high risk older people. This screening tool is a straightforward 11 item self-administered survey which has demonstrated high sensitivity and specificity [26, 27]. In addition, a large cluster randomised controlled trial found that BRIGHT screening is effective in identifying older adults with disability [6]. Each of the 11 questions has a “yes” or “no” answer and represents one count. The scores are summed with a total of three or higher indicating high risk of health and/or functional decline. The BRIGHT screen was posted by the GNS, with a return self-addressed paid envelope, to older people who met eligibility criteria in the intervention group. If the posted BRIGHT screen was not completed and returned within two weeks, the GNS was to undertake follow-up phone calls to administer the BRIGHT over the phone. Subsequently, within one month of receiving the returned BRIGHT all older people deemed high risk (BRIGHT score of 3 or greater) were visited by the GNS in their own home where a CGA was undertaken. The healthcare practice was informed of older people who were identified as high risk due to the BRIGHT screen result. GPs within the healthcare practice could also directly refer older people they were concerned about to the GNS for assessment.

The CGA was to be undertaken within two weeks of the returned BRIGHT screen, in the older person’s home at a mutually convenient time. This assessment comprehensively evaluated the individuals’ body systems including respiratory, cardiac, neurological, gastrointestinal, musculoskeletal and bladder/bowel function. In addition, pain, medications and potential social issues were examined. To assess functional ability the Barthel Index [28] and Lawton Instrumental Activities of Daily Living scale [29] were used. If the possibility of cognitive impairment or depression was identified the Addenbrooke’s Cognitive Examination Revised New Zealand Version (ACE-R) [30] and Brief Assessment Schedule Depression Cards (BASDEC) [31] were utilised. Following this assessment, the GNS provided a summary of the older person’s current issues and developed an individualised intervention plan, this varied depending on personalised need, although often included education, referrals and ongoing GNS input and follow up in the form of phone calls or additional home visits. This summary was sent to the healthcare practice.

The GNS was located within the primary healthcare organisation as well as integrating with hospital based specialist gerontology teams. These specialist teams provided the GNS with mentorship and increased expertise through peer clinical education sessions and weekly case conferences. The combination of primary and secondary care engagement afforded the GNS access to primary healthcare practice and hospital patient databases as well as the ability to simultaneously coordinate with community and specialist hospital services. In addition, the GNS was completing her Masters in Nursing at the time of this study.

### Comparison group

To determine the three comparison health care practices, a list of all healthcare practices within the same PHO were identified. The three practices which were most closely aligned to the intervention group in terms of socioeconomic status, ethnicity and geographical location were selected. Data was collected from a large centralised database meaning no direct contact was needed with older participants in the comparison group. The comparison group received usual care through their primary healthcare practice, this did not involve any type of screening for frailty or the intensive care management provided by a highly trained GNS which the intervention group received. If the GP thought GNS input was required, they would be referred to the hospital based GNS team as per usual referral and practice procedures.

### Data collection

Older people in the intervention group who met the inclusion criteria were posted BRIGHT screens in a phased manner between October 2010 and August 2012. During this period, the BRIGHT screens were posted to potential participants at five time points based on surname. The intervention date coincided with the BRIGHT screen postal date per individual. The comparison group was identified through district health board datasets and matched the criteria used for the postal BRIGHT questionnaire population. For the comparison group, the median date for the five postal time points was calculated.

Data pertaining to hospital outcomes were collected one year pre and one year post the intervention period; one year prior to the start of the intervention (commencing October 2009) and one year post the end of the intervention (concluding August 2013). Data were extracted from an electronic records system. This system contained data on a range of information including demographic details (date of birth, gender, ethnicity), hospital admissions, ED presentations, and length of stay. In New Zealand, all individuals who receive healthcare are assigned a unique National Health Index (NHI) number. This unique number is stored in the National Health Index (NHI) together with the individual’s demographic details. The NHI and individual NHI numbers are used for planning, coordinating and provision of health care services nationwide [32]. This unique NHI data was linked to routinely recorded hospital data for each individual.

### Outcome measures

Healthcare utilisation outcomes for older people in the intervention group were compared with older people in the comparison group. The primary outcome was hospital admissions (defined as acute admissions; assessment, treatment and rehabilitation as well as elective admissions were excluded from analysis). Secondary outcomes were hospital re-admissions (defined as acute admission, after 30 days of discharge, and to the same hospital), ED presentations, length of stay, residential care admissions and community services contacts. Hospital re-admissions were classified as either less than three admissions in a year or three or more admission in a year. This aligned with previous research which defined three or more admissions in a year to be frequent re-admissions [33].

### Data analysis

Data was automatically extracted from an electronic records system into a Microsoft® Excel database which was then imported to SAS/STAT® software for analysis. The data were summarised using descriptive statistical methods detailing frequency counts, percentages and mean scores as appropriate. Student’s *t* tests were used for comparisons of continuous data (including comparisons of age, hospital length of stay, mean hospitalisations, and mean ED presentations) and Chi-square tests for comparisons of categorical data. Relative risks and 95% confidence intervals were calculated for the secondary outcomes (mortality, residential care admission and community contacts) when comparing the intervention and comparison groups.

## Results

**Fig. 1:**
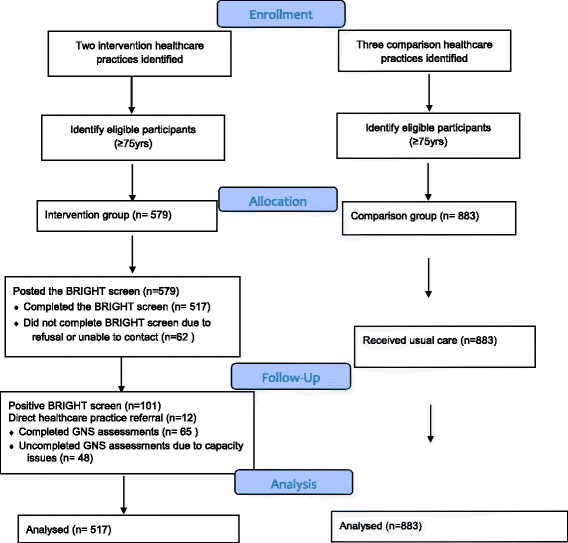
Flow diagram illustrating participant enrolment and progression throughout the study. This file contains the Fig. 1 flow diagram

Figure 1 presents a flow diagram of participant enrolment and progression for the intervention and comparison groups. A total of 579 BRIGHT questionnaires were posted to intervention participants with 517 completed (89% response rate). Of the 517 completed, 20% (*n* = 101) answered ‘yes’ to three or more questions of the 11 item tool which indicated high risk of functional or health decline. The GNS completed a CGA on 65 participants which included 53 participants identified through screening and 12 participants identified through direct referral from the primary healthcare team. Forty-eight were not assessed by the GNS as there was not capacity for one GNS to complete these assessments within the study period. It is important to note that the intervention model continued beyond this study timeframe and all identified high risk older people were assessed.

The comparison group (*n* = 883) mean age (81.9 years) was higher than the intervention group (80.2 years, *p* < 0.0001) and there were more females in the intervention than comparison group, although, this was not significantly different (Table 1). European ethnicity made up the vast majority of participants for both groups. There were no significant differences in baseline characteristics for intervention participants that were or were not assessed by the GNS. Participants identified as ‘high risk’ by scoring a positive BRIGHT screen, were older (mean 82.9 years) and with a higher proportion of females (68%) than the total population screened (Table 2).

**Table 1:** Total screened population: Intervention and comparison group baseline characteristics

	Comparison Group (*n* = 883)	Intervention Group (*n* = 517)	*p*-value
Age, mean (SD)	81.9 (5.0)	80.2 (5.1)	< 0.0001
Gender, *n* (%)			
Female	485 (54.9%)	301 (58.2%)	
Male	398 (45.1%)	216 (41.8%)	0.23
Ethnicity, *n* (%)			
European	773 (87.5%)	491 (95.0%)	
Asian	60 (6.8%)	3 (0.6%)	
Maori	7 (0.8%)	1 (0.2%)	
Pacific Island	3 (0.3%)	–	
Other^a^	40 (4.5%)	22 (4.3%)	< 0.0001

^a^Other: not specified, don’t know

**Table 2:** Positive BRIGHT screen participants baseline characteristics: Intervention group

	Positive BRIGHT (*n* = 101)	GNS assessed^a^ (*n* = 65)	Not GNS assessed (*n* = 48)
Age, mean (SD)	82.9 (4.9)	81.2 (5.7)	83.5 (5.2)
Gender, *n* (%)			
Female	69 (68.3%)	41 (63.1%)	34 (70.8%)
Male	32 (31.7%)	24 (36.9%)	14 (29.2%)
Ethnicity, *n* (%)			
European	97 (96.0%)	63 (96.9%)	45 (93.8%)
Maori	1 (1.0%)	–	1 (2.1%)
Other^a^	3 (3.0%)	2 (3.1%)	2 (4.2%)
Pacific Island	–	–	–
Asian	–	–	–

^a^Other: not specified, don’t know

### Main outcome

Overall, there were no significant differences found in hospitalisations between intervention and comparison groups. Both intervention and comparison groups showed increased hospitalisation over time. There were fewer mean hospitalisations for the comparison group than intervention post-intervention although this was not significantly different.

### Secondary outcomes

There were no significant differences found in emergency department (ED) presentations between intervention and comparison groups and both groups showed increased ED presentations over time. There were lower mean ED presentations for the intervention group than the comparison group (non-significant). Those with frequent hospital re-admissions (three or more) were lower for the intervention group compared to the comparison group. Overall, the hospital length of stay was longer for the intervention group (see Table 3).

**Table 3:** Healthcare utilisation outcomes for comparison and intervention groups

	Comparison Group *n* = 883			Intervention Group *n* = 517			Mean difference (95% CI), *p*-value
Outcome	Baseline	Post intervention	Difference baseline and post intervention	Baseline	Post intervention	Difference baseline and post intervention	
Total hospitalisations (mean)	344 (0.39)	401 (0.45)	57 (0.06)	186 (0.36)	260 (0.50)	74 (0.14)	0.08 (−0.41, 0.92), 0.63
Total ED presentations	364	459	95	186	254	68	
Mean ED presentations	0.41	0.52	0.11	0.36	0.49	0.13	0.02 (−1.58 to 5.77), 0.26
At least one hospital re-admission in 12 months	68 (7.7%)	115 (13%)	5.3%	31 (6%)	64 (12.4%)	6.4%	
< 3 hospital re-admissions in 12 months	59 (6.7%)	86 (9.7%)	3%	30 (5.8%)	54 (10.4%)	4.6%	1.6% (−0.55, 4.02), 0.13
≥ 3 hospital re-admissions in 12 months	9 (1.0%)	29 (3.3%)	2.3%	1 (0.2%)	10 (1.9%)	1.7%	−0.6% (−1.17,-2.17), 0.45
Hospital length of stay,[days] mean (SD)	4.7 (5.3)	5.8 (7.3)	1.1	5.0 (6.4)	6.8 (16.0)	1.8	0.7 (−0.53, 1.93), 0.26

There was higher mortality for the intervention group compared to the comparison group (RR 1.43, CI 0.87 to 2.33, *p* = 0.16), more residential care admissions for the comparison group (RR 0.80, CI 0.46 to 1.38, *p* = 0.41), but fewer district nursing contacts and social work contacts for the intervention group (Table 4). None of these results were significantly different. Conversely, there were greater physiotherapy, occupational therapy and gerontology nurse specialist, dietician, and speech/language therapy contacts for the intervention group than the comparison group, but only physiotherapy contacts were significantly different (*p* = 0.03).

**Table 4:** Mortality, residential care and community contacts for comparison and intervention groups

Outcomes	Comparison Group *n* = 883	Intervention Group *n* = 517	Relative Risk	Confidence Interval	*p*-value
Mortality	33 (3.7%)	28 (5.4%)	1.43	0.87 to 2.33	0.16
Residential care	39 (4.4)	18 (3.5%)	0.80	0.46 to 1.38	0.41
Community services contacts					
District Nurse	41 (4.6%)	21 (4.1%)	0.88	0.53 to 1.47	0.63
Physiotherapy	21 (2.4%)	24 (4.6%)	1.91	1.07 to 3.40	0.03
Occupational therapy	8 (0.9%)	10 (1.9%)	2.11	0.84 to 5.32	0.11
Gerontology CNS^a^	5 (0.6%)	5 (1.0%)	1.70	0.49 to 5.85	0.40
Respiratory CNS^a^	3 (0.3%)	4 (0.8%)	2.27	0.51 to 10.09	0.28
Social work	4 (0.5%)	–			
Dietetics	1 (0.1%)	3 (0.6%)	5.1	0.53 to 48.90	0.16
SLT^b^	1 (0.1%)	1 (0.2%)	1.71	0.11 to 27.23	0.71
Ostomy nurse	–	1 (0.2%)			
Continence Nurse	–	1 (0.2%)			

^a^*CNS* clinical nurse specialist

^b^*SLT* speech language therapy

## Discussion

This study evaluated the impact of a systematic case-finding intervention for community-dwelling older people using a pragmatic controlled before-after evaluation. Healthcare utilisation outcomes were compared for those in the intervention primary healthcare practices with participants receiving usual care in primary healthcare practices one year before and after the start of the intervention. Both groups showed increased hospitalisation over time, possibly indicating declining health over time. Overall, there were no significant differences in the primary outcome of hospitalisation or secondary outcomes of ED presentations, mean hospital length of stay and hospital re-admissions. Comparably, a recent study of a case-finding and nurse-led intervention in primary care also found no statistical difference in hospital admissions, ED presentations or mortality when comparing the intervention and usual care groups. However, a small but statistically significant improvement in daily functioning was found [34]. Nevertheless, our findings are worth further exploration. There were more mean ED presentations compared to hospital admissions for the comparison group post intervention, but fewer for the intervention group suggesting fewer ED presentations led to hospital admission. Furthermore, the data suggested the intervention group had fewer frequent re-admissions (three or more) than the comparison group. These results may indicate that early identification led to more appropriate care, although, the possibility that these differences are a chance result cannot be ruled out.

The model of care implemented in this study was innovative in using a GNS that was integrated across secondary care geriatrics specialist care. It has been recognised that the current primary health model of care is insufficient for the growing population of complex older people and that integration across healthcare settings is vital for complex care older people require [35]. Although integrated care for complex older adults is gaining traction, the research results have been mixed and equivocal. There is mounting international evidence that providing comprehensive assessment, care planning and on-going care coordination not only improves overall quality of care for high risk older adults, but is cost effective by maintaining wellness and decreasing hospital utilisation. In a recent large observational study, team-based care was shown to improve care quality measures, and decrease emergency department admissions and to be more cost effective than traditional care [36]. The GRACE model utilised a nurse practitioner and social worker to care for high risk older people and found significant decrease in acute hospitalisations but only for those at high risk [37].

Our study found intervention participants had more contact from community rehabilitation services such as physiotherapy (almost twice more likely than the comparison group), occupational therapy and speech/language therapy. However, there were less district nurse and social work contacts compared to the comparison group. It may be that the GNS intervention increased referrals to rehabilitation services, while providing some of the services during the intervention that would normally have been supplied through community social work or district nursing services. In the absence of a formal power calculation and adjustment for multiple testing it is unclear if these differences are a chance result and findings may be significantly different with a larger sample. A systematic review of integrated care for high needs older people was inconclusive and more research is needed regarding use of community services for this type of intervention [38]. The findings from this study may represent that community dwelling older people with higher needs could be managed with the help of the GNS and the integrated team rather than needing admission to a long term residential care facility.

Another study using the BRIGHT screen found that those older people identified as high risk for health decline and highlighted to the healthcare practice team showed a smaller decrease in quality of life measures. Further, there was no difference in hospitalisations, although, there was an increase in admission to long-term residential care [6]. These results are contrary to an interesting point estimate in our study that showed the intervention group had a lower risk for long-term care admissions (RR 0.80). This disparity may be the result of identifying high risk without the availability of an expert clinician to provide the subsequent necessary care coordination. It is known that the highest risk for hospitalisation is in the six months prior to admission to long term residential care [39].

The skill of the nurse is important as evidenced in a systematic review of discharge interventions for chronically ill or frail older patients [40]. In the model of care investigated in this study, the skills of the GNS were embedded into the primary healthcare team. The GNS increased integration of care across settings through systematic liaison with the specialist hospital geriatrics team. The level of specialist skills possessed by the GNS has been shown to be essential, with other lower level nursing options, although initially cheaper, not cost-effective long term [41, 42].

As with all research, this study has a number of limitations. The two intervention healthcare practices volunteered to implement this new model of care, therefore, randomisation of healthcare practices was not possible. It is envisaged that in the future, findings from this study would be used to inform a larger randomised controlled trial. Given the absence of randomisation in this study, findings may not be generalizable to larger more diverse populations. All identified high risk older people were unable to receive a CGA within the study timeframe as only one GNS was employed and completing all assessments for qualifying participants within the study timeframe was beyond the GNS capacity. Nevertheless, the fact that not all of the identified high risk older people were assessed should not have introduced a selection bias as the older people assessed were determined based on surname, not functional ability. There was no adjustment for multiplicity in the analysis, therefore, the results of this study may be due to a chance findings. Finally, replication of this innovative model using more than one GNS may elicit different results due to variation.

## Conclusion

New models of healthcare are required that incorporate the gerontology specific needs for the rapidly ageing population. This is especially true in primary healthcare. Identification of high risk older people and intervention by a specialist gerontology nurse embedded in primary healthcare practices is one way of providing a care coordination approach. This study did not include patient reported outcomes, such as quality of life, however, this would be of benefit for future studies. This study demonstrates that this new type of care model is feasible, but more research is needed to determine if the findings demonstrated would show significant differences in a larger study.

## Additional files

Additional file 1: This file contains the completed CONSORT checklist. (DOCX 36 kb)

Additional file 2: This file contains the completed TIDieR checklist. (DOCX 28 kb)

## Abbreviations

BRIGHT: Brief Risk Identification for Geriatric Health Tool
CGA: Comprehensive Geriatric Assessment
CONSORT: Consolidated standards of reporting trials
ED: Emergency department
GNS: Gerontology Nurse Specialist
GP: General practitioner
NHI: National Health Index
PHO: Primary healthcare organisation

## References

[1] De Almeida Mello J, et al. Evaluations of home care interventions for frail older persons using the interRAI home care instrument: a systematic review of the literature*.* J Am Med Dir Assoc, 2015. 16(2): p. 173.e1–173173.e10. 10.1016/j.jamda.2014.11.007 25512214

[2] Gijsen R (2001). Causes and consequences of comorbidity: a review. J Clin Epidemiol.

[3] Popejoy LL (2015). Comparing Aging in Place to Home Health Care: Impact of Nurse Care Coordination On Utilization and Costs. Nursing Economic$.

[4] Tao H, McRoy S (2015). Caring for and keeping the elderly in their homes. Chin Nurs Res.

[5] Boeckxstaens P, De Graaf P (2011). Primary care and care for older persons: position paper of the European forum for primary care. Qual Prim Care.

[6] Kerse N (2014). The cluster-randomized BRIGHT trial: proactive case finding for community-dwelling older adults. The Annals of Family Medicine.

[7] O’Caoimh R (2015). Risk prediction in the community: a systematic review of case-finding instruments that predict adverse healthcare outcomes in community-dwelling older adults. Maturitas.

[8] Boult C (1998). Identification and assessment of high-risk seniors. Am J Manag Care.

[9] Morgan S, Yoder LH (2012). A concept analysis of person-centered care. J Holist Nurs.

[10] Prokop J (2016). Care coordination strategies in reforming health care: a concept analysis. Nurs Forum.

[11] Yang YT, Meiners MR (2014). Care coordination and the expansion of nursing scopes of practice. Journal of Law, Medicine & Ethics.

[12] Coleman EA (2006). The care transitions intervention: results of a randomized controlled trial. Arch Intern Med.

[13] Corbett HM (2005). Care coordination in the emergency department: improving outcomes for older patients. Aust Health Rev.

[14] Naylor MD (2004). Transitional care of older adults hospitalized with heart failure: a randomized, controlled trial [corrected] [published erratum appears in J AM GERIATR SOC 2004 Jul;52(7):1228]. J Am Geriatr Soc.

[15] Stuck AE (1993). Comprehensive geriatric assessment: a meta-analysis of controlled trials. Lancet.

[16] Ellis G (2011). Comprehensive geriatric assessment for older adults admitted to hospital. Cochrane Database Syst Rev.

[17] DeFlon S (2012). The role of the clinical nurse specialist as clinician and advocate in a primary health care clinic. Creative Nursing.

[18] Roberts J, Floyd S, Thompson S (2011). The clinical nurse specialist in New Zealand: how is the role defined?. Nursing Praxis In New Zealand Inc.

[19] Vestjens L, Cramm JM, Nieboer AP (2016). An evaluation of an integrated primary care approach to improve well-being among frail community-dwelling older people. International Journal of Integrated Care (IJIC).

[20] Nicholson C (2016). Integrated primary/secondary health care governance: we have the evidence, do we have the practice?. International Journal of Integrated Care (IJIC).

[21] Savage E (2016). Transforming chronic illness management through integrated care: a systematic review of what works best and why. International Journal of Integrated Care (IJIC).

[22] King A, Boyd M, Dagley L (2017). Use of a screening tool and primary health care gerontology nurse specialist for high-needs older people. Contemporary Nurse: A Journal for the Australian Nursing Profession.

[23] King AII, et al. Implementation of a gerontology nurse specialist role in primary health care: health professional and older adult perspectives. J Clin Nurs. 2017; 10.1111/jocn.14110 29052288

[24] Hoffmann TC, et al. Better reporting of interventions: template for intervention description and replication (TIDieR) checklist and guide. BMJ (Clinical Research Ed). 2014;348:g1687–7. 10.1136/bmj.g1687 24609605

[25] Ministry of Health. *Primary health care*. 2017 [cited 2017 9 December]; Available from: http://www.health.govt.nz/our-work/primary-health-care.

[26] Boyd M (2008). Emergency department case-finding for high-risk older adults: the brief risk identification for geriatric health tool (BRIGHT). Acad Emerg Med.

[27] Kerse N (2008). The BRIGHT tool. Age & Ageing.

[28] Mahoney FI, Barthel DW (1965). Functional evaluation: the BARTHEL index. Maryland State Medical Journal.

[29] Lawton MP, Brody EM (1969). Assessment of older people: self-maintaining and instrumental activities of daily living. The Gerontologist.

[30] Mioshi E (2006). The Addenbrooke's cognitive examination revised (ACE-R): a brief cognitive test battery for dementia screening. International Journal of Geriatric Psychiatry.

[31] Adshead F, Cody DD, Pitt B. BASDEC: a novel screening instrument for depression in elderly medical inpatients. BMJ (Clinical Research Ed.). 1992;305(6850):397–7. 10.1136/bmj.305.6850.397 PMC1883136 1392921

[32] Ministry of Health. *National Health Index*. 2017 [cited 2017 9 December]; Available from: http://www.health.govt.nz/our-work/health-identity/national-health-index.

[33] Kirby SE (2010). Patient related factors in frequent readmissions: the influence of condition, access to services and patient choice. BMC Health Serv Res.

[34] Bleijenberg N (2016). Effectiveness of a proactive primary care program on preserving daily functioning of older people: a cluster randomized controlled trial. J Am Geriatr Soc.

[35] Rowe JW, Fulmer T, Fried L (2016). Preparing for better health and health Care for an Aging Population. JAMA.

[36] Reiss-Brennan B (2016). Association of Integrated Team-Based Care with Health Care Quality, utilization, and cost. JAMA.

[37] Counsell SR (2009). Cost analysis of the geriatric resources for assessment and care of elders care management intervention. J Am Geriatr Soc.

[38] Hopman P (2016). Effectiveness of comprehensive care programs for patients with multiple chronic conditions or frailty: a systematic literature review. Health Policy.

[39] Boyd M (2016). Hospitalisation of older people before and after long-term care entry in Auckland, New Zealand. Age & Ageing.

[40] Allen J, et al. Quality care outcomes following transitional care interventions for older people from hospital to home: a systematic review. BMC Health Serv Res. 2014;14:346–6. 10.1186/1472-6963-14-346 PMC4147161 25128468

[41] Brooten D (2002). Lessons learned from testing the quality cost model of advanced practice nursing (APN) transitional care. J Nurs Scholarsh.

[42] Bryant-Lukosius D (2016). Framework for evaluating the impact of advanced practice nursing roles. J Nurs Scholarsh.

